# Current Trends in the Management of Hiatal Hernia: A Literature Review of 10 Years of Data

**DOI:** 10.7759/cureus.71921

**Published:** 2024-10-20

**Authors:** Vinod Kumar Singhal, Adil Md Suleman, Nufra Senofer, Vidher VV Singhal

**Affiliations:** 1 Department of General Surgery, PRIME Hospital, Dubai, ARE; 2 Department of Ear, Nose, and Throat (ENT), PRIME Hospital, Dubai, ARE; 3 College of Medicine, University College London, London, GBR

**Keywords:** hiatal hernia, laparoscopic, linx, nissen, open surgery, toupet

## Abstract

Hiatal hernia (HH) is commonly detected during endoscopic examinations and is associated with gastroesophageal reflux disease. In recent years, there have been significant advancements in diagnosing and treating HH. Surgical techniques for HH repair include open surgery, various laparoscopic procedures, transoral incisionless fundoplication, and magnetic sphincter augmentation (MSA). Laparoscopic Nissen fundoplication is often considered the standard for treating gastroesophageal reflux disease-related HH due to its effectiveness. Other procedures, such as Toupet and Dor fundoplications, may be suited for patients with specific conditions, such as impaired esophageal motility. Newer approaches, including the MSA system and mesh repair, focus on patient-specific treatments to achieve the best outcomes. This review synthesizes the literature from 2014 to 2024 to provide an overview of current trends in HH management.

## Introduction and background

Since hiatal hernia (HH) is frequently observed during endoscopic examinations, with a reported prevalence of 20%, it is considered a typical variation rather than a pathological condition [1]. HH is a prevalent condition in which the stomach or other abdominal organs protrude through the esophageal hiatus of the diaphragm into the thoracic cavity [2]. It is strongly linked to gastroesophageal reflux disease (GERD) and can cause various symptoms such as heartburn, regurgitation, difficulty swallowing (dysphagia), and chest pain [2,3]. HH occurs due to elevated pressure within the abdomen, causing the stomach and other abdominal organs to protrude into the mediastinum [2,4]. The primary risk factors for its development are being overweight or elderly [5]. Other acknowledged risk factors include multiple pregnancies, a history of esophageal surgery, partial or total gastrectomy, and specific skeletal system disorders related to bone decalcification and degeneration [5,6]. Over the past 10 years, there have been considerable advancements in managing HH, especially in diagnosis and surgical treatment. The diagnostic approach has remained mostly consistent, utilizing imaging techniques such as barium swallow, endoscopy, and high-resolution manometry to evaluate the size, location, and type of HH [2,3].

The current anatomical classification of HHs comprises four types. Type I, or sliding hernias, are the most common form. In these cases, a weakness in the phrenoesophageal ligament permits the gastroesophageal junction to herniate into the thoracic cavity, causing the cardia of the stomach to move above the diaphragmatic hiatus. Type II, a paraesophageal hernia, occurs when the gastroesophageal junction stays in place. At the same time, another part of the stomach protrudes through the diaphragmatic hiatus into the chest next to the esophagus. Type III hernias are a combination of both type I and type II. Type IV HH indicates the herniation of an intra-abdominal organ, typically the colon or small bowel, alongside the stomach through the hiatus. However, it may also involve organs like the spleen or pancreas [7]. The Society of American Gastrointestinal and Endoscopic Surgeons (SAGES) guidelines provide specific recommendations for each type of HH, as the indications and treatments vary between axial (type I) and para esophageal hernias (PEH) (types II, III, and IV). According to these guidelines, “the primary clinical importance of a type I HH lies in its association with GERD” [2]. The European Association of Endoscopic Surgery (EAES) guidelines for managing GERD recommend laparoscopic antireflux surgery for patients who experience a persistently reduced quality of life, continuous troublesome symptoms, and disease progression despite appropriate proton pump inhibitor (PPI) therapy in both dosage and usage [8]. All symptomatic patients with paraesophageal HHs (types II, III, and IV) should undergo surgical repair [1].

Several techniques for managing HHs have been used over the past decade [9], including open surgery, laparoscopy (Nissen fundoplication, Toupet fundoplication, Dor fundoplication), transoral incisionless fundoplication (TIF), magnetic sphincter augmentation (MSA) (LINX; Torax Medical, Inc., Shoreview, USA), and other medical approaches (lifestyle modifications, medications). Among these techniques, two technical aspects that could impact the outcome are still under debate: mesh-augmented cruroplasty and choosing between a 360-degree Nissen fundoplication and a 270-degree Toupet fundoplication [10]. Some literature suggests that similar outcomes to those of Nissen and Toupet fundoplication can be achieved with DOR anterior hemifundoplication as an alternative [11]. TIF is a minimally invasive endoscopic procedure designed to reposition the distal esophagus below the diaphragm. This technique establishes a high-pressure zone similar to surgical fundoplication but with fewer anatomical alterations [12]. Unlike traditional anti-reflux surgery, it is performed entirely through the mouth without surgical incisions. TIF provides a treatment option for patients who have not responded well to PPI medications or wish to avoid long-term use of these drugs and their potential side effects [13,14].

Recently, two novel treatment options for managing axial HH with GERD have emerged. The LINX system uses magnetic beads to augment the esophageal sphincter, and the EndoStim system employs electrical stimulation to enhance the function of the lower esophageal sphincter (LES) [15,16]. Comparative studies have indicated that the quality of life for individuals with paraesophageal hernias remains similar regardless of whether they undergo fundoplication. Therefore, fundophrenicopexy is an alternative to fundoplication in cases of more severe para esophageal HHs without reflux symptoms [17]. This review aims to synthesize the literature from the past 10 years to provide a comprehensive overview of current trends in managing HH.

## Review

Methodology

Study Design and Strategies

**Figure 1:**
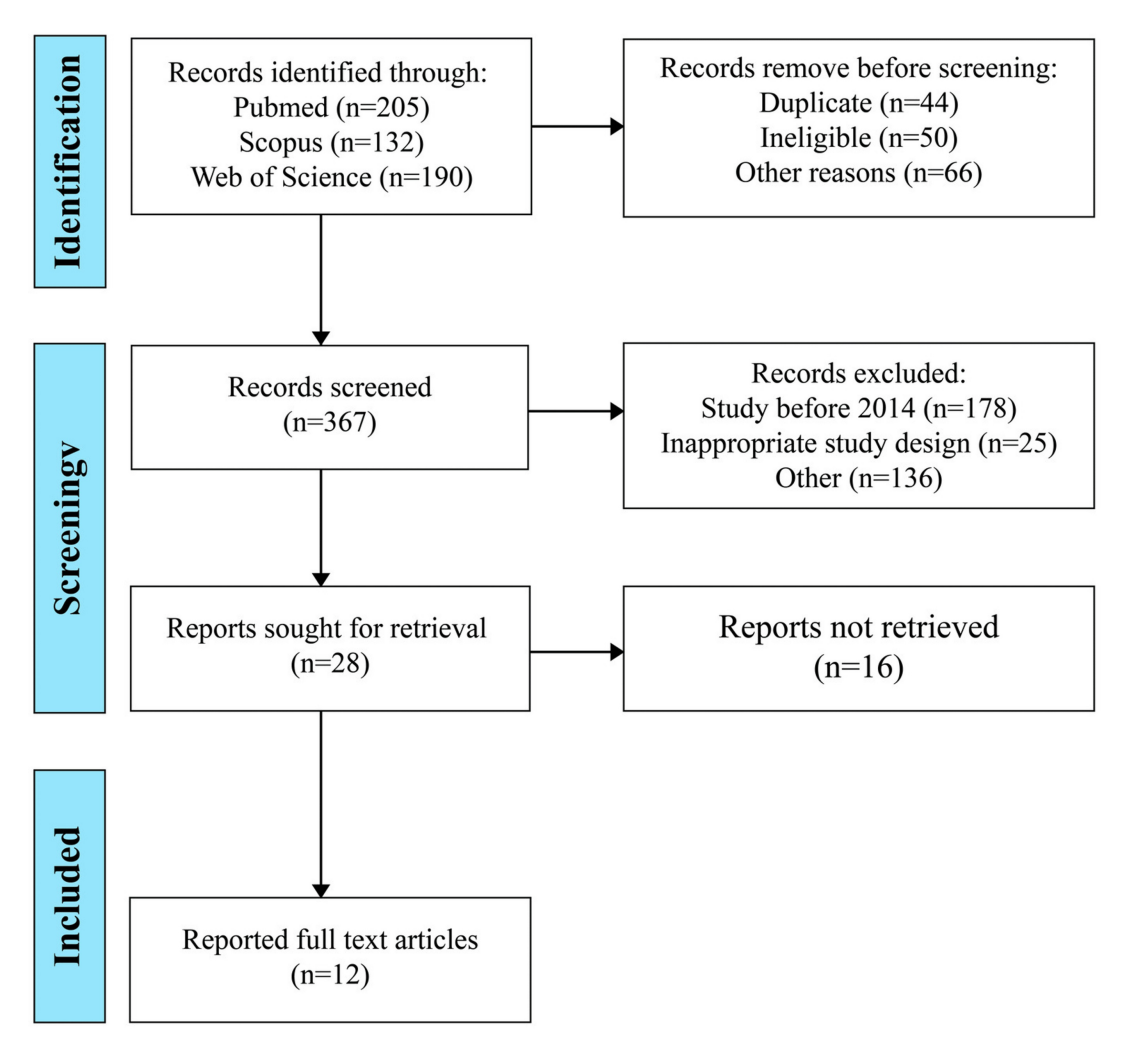
PRISMA chart PRISMA: Preferred Reporting Items for Systematic Reviews and Meta-Analyses

The study was analyzed using a systematic review methodology, focusing on peer-reviewed articles published over the last decade (Figure 1). We searched databases such as PubMed, Scopus, and Web of Science, spanning 2014 through May 2024, using keywords like "hiatal hernia management" and "current trends." Inclusion criteria were set to select studies that addressed advancements in diagnostic techniques, surgical interventions, and postoperative outcomes. Data were extracted and categorized based on publication year, study design, sample size, and critical findings.

Inclusion Criteria

Studies included observational research designs, such as cross-sectional, cohort, and case-control studies, that address HH management and studies that evaluated treatment outcomes, encompassing disease severity and specific characteristics.

Exclusion Criteria

Articles published before 2014, reviews, meta-analyses, case series, case reports, animal studies, and non-English language studies were excluded from the analysis.

Data Extraction

Two reviewers independently gathered and compiled pertinent data from the selected studies, ensuring a thorough extraction process. Information extracted included author identification (names and publication years), study design, population characteristics (sample size), interventions, and critical findings. The main outcome of this systematic review and meta-analysis is to provide a comprehensive overview of the current trends in managing HHs for the past 10 years. Any discrepancies in the data extraction process were reconciled by consulting a third reviewer, further ensuring the accuracy and completeness of our data. This review incorporates studies published in English up to May 2024 from the inception of relevant databases for consistency and comprehensive analysis.

Statistical Analysis

Our study utilized robust statistical analysis to analyze the current trends in managing HH over the past decade, ensuring objectivity and scientific rigor. Data extraction tables were used to summarize the key findings of all eligible studies, recorded using Microsoft Excel 2021 (Microsoft, Redmond, WA, USA). Studies were categorized based on the prevalence and outcomes of different surgical techniques for HH repair, including open surgery, laparoscopic approaches, various types of fundoplication (Nissen, Toupet, and Dor), TIF, and MSA (LINX).

Results and discussion

Table 1 represents the summary of selected studies [18-26] while Table 2 represents treatment procedures for HH [27-36].

**Table 1:** Summary of different types of treatment of hiatal hernia GERD: gastroesophageal reflux disease

Recent Reference	Year	Description	Advantages	Disadvantages
Sfara and Dumitrascu [3]	2019	Weight loss, elevating the head of the bed, avoiding meals before bedtime, eliminating trigger foods.	Non-invasive, first line of management.	It requires patient adherence, but may not be sufficient for severe cases.
Yu et al. [5]	2018	Used for moderate symptoms; it can be on-demand or add-on treatment.	Provides symptom relief for GERD.	It may not be effective for persistent symptoms and is not a definitive treatment for paraesophageal hernias.
Köckerling et al. [10]	2020	Fixation of the stomach fundus to the diaphragm is often used with other techniques.	It secures the stomach in a normal position and is effective for large or complex hernias.	It is unsuitable for all hiatal hernias, so additional surgical techniques are required.
Hosein et al. [18]	2021	Involves a large incision; the stomach is pulled back into the abdominal cavity, and the fundus is wrapped around the lower esophagus to prevent acid reflux.	Direct access for surgeons allows for extensive repairs.	Higher risk compared to laparoscopic repair, higher morbidity rate, longer hospitalization, and more postoperative pain.
Yano et al. [19]	2021	Minimally invasive surgery using small incisions and a video monitor.	Improved visualization, shorter hospital stays, less postoperative pain, and decreased morbidity.	Two-dimensional imaging, limited motion of instruments, poor ergonomics, and higher recurrence rates.
Jaruvongvanich et al. [20]	2023	360-degree wrap of the stomach around the lower esophagus.	The gold standard for GERD treatment: stops all reflux and fixes the hiatal hernia simultaneously.	Long-term side effects include gas bloat, inability to belch or vomit, and potential anatomic failure.
Ugliono et al. [21]	2022	270-degree wrap of the stomach around the lower esophagus.	Effective for GERD, preserves swallowing better than Nissen; less postoperative dysphagia and gas bloat.	Uncertainty regarding long-term durability, with mixed study results.
Trepanier et al. [22]	2019	180-degree wrap of the stomach around the anterior aspect of the esophagus.	Less invasive compared to full fundoplications, preserves swallowing function.	Higher chance of recurrent symptoms.
Watkins et al. [23]	2018	Use of biologic or synthetic mesh to reinforce the hiatal defect.	Reduces risk of hernia recurrence, tension-free repair.	Complications include mesh erosion and increased difficulty for revision surgery.
Vasudevan et al. [24]	2018	Uses the DaVinci system for enhanced visualization and ergonomics.	Enhanced 3D visualization, improved ergonomics, effective and safe with low complication rates.	Higher cost and lack of large randomized trials comparing outcomes with laparoscopic surgery.
Bologheanu et al. [25]	2022	Placement of a flexible ring of magnets around the lower esophagus to prevent reflux.	Augments physiological barriers to reflux, does not alter gastric anatomy, reversible, and highly successful.	Device size and higher cost.
Oppenheimer et al. [26]	2020	An eight-week course is recommended for GERD symptom relief, with the minimal effective dose advised.	Can be used in varying dosages to reduce gastric acid secretion.	Long-term use may have side effects and may not be effective for paraesophageal hernias.

**Table 2:** Treatment procedures for hiatal hernia based on years TIF: transoral incisionless fundoplication

Authors	Year	Nissen Fundoplication	Toupet Fundoplication	Dor Fundoplication	TIF	LINX
Jaruvongvanich et al. [20]	2023	70	-	-	125	-
Trepanier et al. [22]	2019	58	-	48	-	-
Su et al. [27]	2016	149	41	86	-	-
Skubleny et al. [28]	2016	-	-	-	-	415
Buckley et al. [29]	2017	-	-	-	-	200
Huerta et al. [30]	2019	117	62	-	-	-
Janu et al. [31]	2019	-	-	-	99	-
Li et al. [32]	2019	61	-	-	-	-
Guan et al. [33]	2021	-	-	152	-	-
Gergen et al. [34]	2022	-	-	-	12	-
Sovpel et al. [35]	2022	109	62	-	-	-
Wu et al. [36]	2022	34	66	-	-	27

This analysis uncovers current trends in HH repair. A scoping review identified nine articles focusing on HH management. Despite the increase in publications over the past decade, the primary emphasis has remained on treatment strategies. Our findings indicate that few papers specifically discuss HH management. The literature reviewed in this study, however, examined the efficacy and evolving trends in HH management over recent decades. These studies reviewed various surgical approaches for treating HH. Surgery for HH repair aims to reduce the size of the hernia sac, restore the normal anatomy of the gastroesophageal junction, and prevent reflux of stomach contents into the esophagus. Several surgical techniques are used, each with advantages and considerations [37].

Open Surgery

Traditional hernia repair entails a larger incision in the abdomen, which exposes the surgeon to more significant risks compared to laparoscopic techniques. The advantages include direct access for surgeons, allowing for extensive repairs [38]. This procedure usually carries more risks than laparoscopic repair in terms of a higher morbidity rate, more extended hospitalization, and more postoperative pain [39,40]. During this procedure, the surgeon repositions the stomach into the abdominal cavity and encircles the upper portion (the fundus) around the lower esophagus to form a snug sphincter, thereby preventing stomach acid reflux. Occasionally, it may be necessary to insert a tube to maintain stomach positioning, which the physicians will remove after a few weeks [41].

Laparoscopic Surgery

Laparoscopy provides enhanced clarity for observing the hiatus, enabling precise dissection of the esophagus and hernia sac, even deep into the mediastinum, all under direct visualization. This method offers several advantages over open repairs, including shorter hospital stays, reduced reliance on nasogastric tubes, diminished postoperative discomfort, and lower morbidity rates [2]. Additionally, laparoscopy offers reduced hospital stays, decreased postoperative pain, and improved aesthetic outcomes. Currently, it is the preferred method for most HH repairs [7].

Fundoplication

Fundoplication involves wrapping the gastric fundus around the esophagus to create a one-way valve that allows food to pass into the stomach but prevents reflux into the esophagus. Fundoplication is a critical surgical procedure to improve postoperative quality of life and alleviate GERD symptoms. It includes several approaches, such as Nissen fundoplication, Toupet fundoplication, and Dor fundoplication.

Nissen Fundoplication: Posterior (360°)

Nissen fundoplication is the foremost choice for treating GERD in individuals whose response to medications is incomplete or who cannot tolerate them for various reasons [42]. The laparoscopic Nissen fundoplication stands out as the leading antireflux procedure, according to Seeras et al. (2023) [43]. Executed through small incisions, laparoscopic procedures allow surgeons to operate while observing via a video monitor [44]. This surgical technique involves wrapping the top of the stomach (known as the fundus) around the lower part of the esophagus, hence the term fundoplication. This wrap effectively corrects the malfunctioning valve at the end of the esophagus, a common issue in GERD patients. Unlike medications, which only partially address GERD symptoms, surgery offers a comprehensive solution by halting the reflux of digestive enzymes and acid.

Consequently, surgical intervention holds the potential to cure reflux, a feat beyond the capabilities of medications alone. Additionally, if an HH coexists, it can be repaired simultaneously with the Nissen fundoplication procedure [42,45]. Nevertheless, this procedure remains underutilized due to concerns about potential long-term complications such as gas retention, difficulty burping or vomiting, and anatomical issues with the repair [43]. A review of studies from the last decade, where surgeons compared Nissen fundoplication for HH with other fundoplication techniques or surgical procedures, found that Nissen fundoplication stands out as an ideal approach. The evidence suggests that Nissen fundoplication is associated with lower complication, mortality, and morbidity rates [46].

Toupet Fundoplication: Posterior (270°)

The Toupet fundoplication offers a partial wrap as opposed to the Nissen fundoplication. While the Nissen procedure fully encircles the lower esophagus with the stomach (a 360-degree wrap), the Toupet procedure involves a 270-degree wrap [47]. In this technique, the surgeon envelops the stomach’s upper portion (fundus) around the lower esophagus and secures it with sutures. This construction forms a valve mechanism, fortifying the LES and halting the reflux of stomach acid into the esophagus. The Toupet fundoplication is particularly effective in addressing GERD symptoms while preserving the ability to swallow more effectively than other fundoplication techniques. Studies have shown that the Toupet technique results in less postoperative dysphagia and gas bloat compared to the Nissen procedure, especially in patients with poor esophageal motility [48,49]. This review found that Toupet fundoplication was used less frequently than Nissen fundoplication (Table 2) [27,30,35]. The main concerns with Toupet fundoplication are the uncertainty regarding its long-term durability and maintaining its effects over time. The reliability and accuracy of meta-analyses comparing Toupet fundoplication with other fundoplication procedures are limited due to the extreme heterogeneity between studies regarding methodological quality, patient characteristics, and surgical techniques. This variability in study designs makes it challenging to draw definitive conclusions on the long-term effectiveness of Toupet fundoplication [21].

Dor Fundoplication: Anterior (180°)

DOR anterior hemifundoplication involves partial stomach wrapping around the esophagus but differs in the direction and extent of the wrap from Toupet fundoplication. The DOR anterior hemifundoplication involves a 180-degree wrap [47]. The DOR anterior hemifundoplication consists of wrapping the stomach’s fundus around the anterior (front) aspect of the esophagus. In contrast, the Toupet fundoplication wraps the fundus around the posterior (back) aspect of the esophagus [42,50]. Studies suggest a higher likelihood of recurrent reflux symptoms with the DOR anterior hemifundoplication. The DOR anterior partial wrap is considered a less durable form of fundoplication, potentially leading to less favorable long-term outcomes than total fundoplications [10,43]. Based on previous studies, DOR could be a good option for HH [22,27-33]. The choice between DOR and Toupet fundoplication depends on surgeon preference, experience, and individual patient factors.

TIF

TIF is an endoscopic procedure designed to reposition the distal esophagus into a subdiaphragmatic location within the stomach. This aims to create a high-pressure zone that mimics the functional and anatomical effects of surgical fundoplication while causing minimal changes to the anatomy of the gastroesophageal junction, fundus, and diaphragmatic hiatus [12]. In the concomitant-TIF (c-TIF) cohort, initial hernia repair was conducted laparoscopically via four ports, with an optional fifth port available for improved retraction and exposure. Hiatal dissection proceeded until 2 to 3 cm of tension-free intraabdominal esophagus was visible [51]. The procedure identified and preserved both vagus nerves. The hiatal defect was then repaired using interrupted posterior sutures to reapproximate the esophageal crura, employing a bougie sized between 48F and 54F, typically 50F, or an endoscope to prevent esophageal constriction. The abdomen was subsequently closed, and patients were positioned in partial left lateral decubitus on a tilted operating table for the c-TIF procedure. The c-TIF 2.0 iteration using the EsophyX device (Endo Gastric Solutions, United States), described by Bell and Cadière and initially published by Jobe et al. (2008), was used for the procedure [52,53]. The valve was created approximately 270 degrees around the esophagus. The TIF procedure is equipped with specialized grippers and fasteners that assist in repairing or reconstructing the valve to enhance its function as a barrier against acid reflux. Unlike traditional open or laparoscopic surgeries, TIF does not require incisions, potentially leading to a faster and less painful recovery. Surgeons have increasingly adopted c-TIF as a treatment modality over the past few years. Jaruvongvanich et al. reported in 2020 that surgeons performed c-TIF more frequently than Nissen fundoplication [20].

MSA (LINX)

The LINX procedure, also known as MSA, is a surgical technique for managing HHs. This procedure involves the placement of a minor, flexible ring of magnets around the lower esophagus to augment the LES and prevent the reflux of stomach contents into the esophagus. The LINX device opens to allow food and liquid down and then closes to prevent stomach contents from moving up, effectively strengthening the sphincter and reducing reflux symptoms [46]. MSA emerged to bridge the treatment gap by introducing a laparoscopic technique that preserves gastric anatomy while enhancing the body’s natural defense against reflux, with the added benefit of reversibility. Engineered for simplicity and reliability, this outpatient procedure focuses on implanting the device. As a result, numerous centers throughout the United States have documented notable success rates and consistently positive clinical results [54,55]. Researchers have identified LINX devices sized 13 or smaller as an independent risk factor for developing dysphagia following surgery. MSA is more expensive than traditional fundoplication techniques [25]. MSA (LINX) offers several advantages in the treatment of HHs, particularly for patients suffering from GERD. One of the primary benefits is that it is a minimally invasive procedure performed laparoscopically, resulting in smaller incisions, reduced scarring, and a faster recovery time compared to traditional surgical methods [29]. Additionally, LINX is highly effective in reducing reflux symptoms by reinforcing the LES, preventing the backward flow of stomach acid. Unlike some other surgical treatments, LINX preserves normal esophageal functions such as swallowing, belching, and vomiting, which are important for maintaining the patient’s quality of life. Moreover, the procedure has a lower risk of complications like gas bloat syndrome, which is often seen after more invasive surgeries. Patients typically experience a quicker recovery with fewer post-operative dietary restrictions, allowing them to return to their normal routines sooner. Importantly, the LINX device is also reversible, providing the option for removal if necessary, making it a flexible and patient-friendly option in the management of HHs [30,36]. The device is also adjustable and removable, providing flexibility for future treatments if necessary. Studies indicate that LINX results in significant symptom relief and improved quality of life with fewer long-term complications than other surgical options like Nissen fundoplication [30,36].

## Conclusions

In conclusion, managing HH has evolved significantly over the past decade, with advancements in diagnostic and treatment modalities. Techniques such as high-resolution manometry have improved the accuracy of detecting and evaluating HHs. Treatment options now include various surgical techniques, such as laparoscopic repairs, and innovative methods like MSA and robotic-assisted surgery. While laparoscopic Nissen fundoplication remains the gold standard for treating GERD associated with HH, alternative procedures like Toupet and Dor fundoplications offer viable options, particularly for specific patient conditions. The integration of TIF and the emergence of the LINX system further expand the therapeutic arsenal. Ultimately, the treatment choice should be individualized, considering patient-specific factors and symptom severity to ensure optimal HH management.
